# Combining fish and environmental PCR for diagnostics of diseased laboratory zebrafish in recirculating systems

**DOI:** 10.1371/journal.pone.0222360

**Published:** 2019-09-12

**Authors:** Manuel Miller, Sibylle Sabrautzki, Andreas Beyerlein, Markus Brielmeier

**Affiliations:** 1 Research Unit Comparative Medicine, Helmholtz Zentrum München, German Research Center for Environmental Health, Neuherberg, Germany; 2 Institute of Computational Biology, Helmholtz Zentrum München, German Research Center for Environmental Health, Neuherberg, Germany; University of Portsmouth, UNITED KINGDOM

## Abstract

Precise knowledge of the health status of experimental fish is crucial to obtain high scientific and ethical standards in biomedical research. In addition to the use of sentinel fish, the examination of diseased fish is a fundamental part of all health monitoring concepts. PCR assays offer excellent sensitivity and the ability to test a broad variety of pathogenic agents in different sample types. Recently, it was shown that analysis of environmental samples such as water, sludge or detritus from static tanks can complement PCR analysis of fish and is actually more reliable for certain pathogens. In our study, we investigated whether the analysis of filtered water mixed with detritus of tanks including fish showing clinical signs of illness is suitable to complement health monitoring programs in recirculating systems. The obtained data indicate that pathogens such as *Pseudoloma neurophilia* or *Myxidium streisingeri* were exclusively or mainly found in fish, while mycobacteria were predominantly present in environmental samples. A combination of both sample types seems to be required for the detection of a broad range of infectious agents in zebrafish colonies using real-time PCR technology.

## Introduction

Reliable detection of infectious pathogens is essential for meaningful health monitoring in experimental fish facilities. The presence of clinical disease or subclinical infection may greatly impact research outcomes and may significantly affect animal health and welfare [1–3]. Several agents have been proven to alter physiological, immunological or behavioral parameters in zebrafish [2]. For example piscine mycobacteriosis, caused by several species of the genus *Mycobacterium*, can lead to broad-ranging manifestations, such as dermal lesions, swollen abdomen, emaciation, non-physiological swimming behavior, and granulomas in almost all tissues. Infections tend to be associated with increased mortality and decreased reproductive output, especially if fish are infected with *M*. *haemophilum* and *M*. *marinum* [4, 5]. Inflammatory changes and an increased incidence of neoplasms of the intestine, such as carcinomas and mixed malignant neoplasms, have been found to be associated with *Pseudocapillaria tomentosa* infection, a common nematode in zebrafish facilities [6]. However, the influence of pathogens that usually cause infections with subclinical course should not be underestimated. These pathogens often remain undetected and little is known about their influence on experimental results so far [4]. For example, the microsporidium *Pseudoloma neurophilia* is known to be one of the most prevalent non-excluded parasites in fish facilities worldwide, and mainly causes subclinical infections in immunocompetent fish. Spagnoli et al. demonstrated that this infection caused changes in shoaling behavior with a potentially wide-ranging impact on neurobehavioral research [7]. Many pathogens included in hygiene monitoring programs are zoonotic, e.g. *Mycobacterium marinum*, which poses a risk to human health [5, 8, 9]. Knowledge of the accurate health status may help to standardize the microbiological quality of animals and therefore to reduce the number of fish used for experiments according to the 3Rs [10]. Precise advice and protocols for fish health monitoring are still extremely rare [3, 11]. A FELASA working group was established to propose a protocol considering agents, test methods, and frequency after reviewing available information on pathogens and their prevalence in different geographical areas and common practices in fish health monitoring [12]. Adapted from rodent monitoring programs, the testing of colony fish or the use of sentinel fish exposed to water from a large number of tanks is quite common. Depending on whether the sentinel fish are used to monitor the health status of the fish or to control the filtration system, either pre-filtration or post-filtration sentinel fish can be used. These animals are examined after an appropriate exposure time as representatives for the complete population of the respective recirculating system [11, 13]. In rodent facilities, the use of environmental samples to complement or completely replace sentinel monitoring has been demonstrated as a secure and sensitive alternative strategy [14–16]. Entire barriers can be reliably monitored via exhaust air dust, with pathogens detected even at very low prevalence [17–20]. For fish, the use of environmental samples such as water and detritus from static tanks or swabs has proven to be a useful method to complement the analysis of sentinel fish [11, 21, 22]. In addition to fish scheduled for routine monitoring, examination of sick animals showing clinical signs of illness constitutes an important part of health surveillance programs. In this study, we compared for the first time real-time PCR analysis of individual diseased zebrafish with environmental real-time PCR of water and detritus to complement our health monitoring program. Over a period of six months, fish showing clinical signs of illness housed in recirculating water systems were collected and euthanized. Detritus and water samples from affected tanks were mixed and filtered for concentration. Both sample types were sent to a commercial diagnostic laboratory and tested using real-time PCR technology, which allows for rapid, sensitive, and cost-effective screening of all life stages of pathogens within frozen fish and environmental samples [23].

## Materials and methods

### Fish and housing

Approximately 9.000 zebrafish (*Danio rerio*) of different genetic backgrounds are kept in three separated barriers of which one is run as the quarantine unit under specified pathogen-free (SPF) conditions. Fish are primarily used for neurological and behavioral research as well as research in developmental biology. The three barriers contain seven individual recirculating water cycles. Stock density is five adult fish per liter. Animals are housed in glass tanks (10 L) obtained from Aqua-Schwarz GmbH (Göttingen, Germany) or plastic Type I mouse cages converted into fish tanks (2.1 L). Facilities are maintained with a 14/10-hour light/dark cycle. Tanks are permanently flooded with reverse osmosis water equipped with sea salt, calcium sulphate, calcium carbonate, and sodium hydrogen carbonate. The waste water is biologically and mechanically filtered and irradiated with UV light before being reintroduced into the tanks. Temperature is set to 26.5° - 27°C. At least 5% but no more than 20% of the total water volume is refreshed automatically per day. The water quality is set to a conductivity of 670–730 μS cm^-1^, pH 6,8–7,0, nitrate <50 ppm, nitrite and ammonia <2 ppm. Depending on the developmental stage, fish are fed with rearing food (JBL NovoTom artemia; JBL GmbH & Co. KG, Neuhofen, Germany), hatched artemia, and commercially available flake food (Tetramin; Tetra GmbH, Melle, Germany) manually distributed by spray bottles two to three times daily. Plastic plants are routinely introduced in mating tanks and exceptionally also in colony tanks to reduce aggression. Live fish are only imported into the quarantine barrier and are transferred into the husbandry facilities via bleached eggs using sodium hypochlorite. Inspection of welfare conditions is performed daily by animal keepers and routinely by competent veterinarians according to a score sheet developed in-house. Access to the fish facility is restricted to experienced animal keepers and researchers by an automated key chip system with quarantine times between the individual barriers. Wearing of gloves and overshoes is mandatory.

### Health monitoring

Routine health monitoring is performed quarterly for each of the seven water cycles by examination of pooled samples of colony animals (mostly escapers from the sump) and fish showing clinical signs of illness. These pooled samples are sent to a specialized diagnostic laboratory for real-time PCR analysis for a panel of selected pathogens including *Edwardsiella ictaluri*, *Flavobacterium columnare*, *Ichthyophthirius multifiliis*, Infectious spleen & kidney necrosis virus (ISKNV), Zebrafish picornavirus (ZfPV-1), *Mycobacterium spp*., *Mycobacterium abscessus*, *Mycobacterium chelonae*, *Mycobacterium fortuitum*, *Mycobacterium haemophilum*, *Mycobacterium marinum*, *Mycobacterium peregrinum*, *Mycobacterium gordonae*, *Mycobacterium saopaulense*, *Myxidium streisingeri*, *Piscinoodinium pillulare*, *Pleistophora hyphessobryconis*, *Pseudocapillaria tomentosa*, and *Pseudoloma neurophilia*. If necessary, animals showing signs of disease are sent to external diagnostic laboratories for further examination. In addition, total bacterial counts of water samples are determined once a year to monitor water quality. For this, water samples from different tanks from each cycle are pooled. A serial dilution on nutrient agar plates following incubation at 20°C and 30°C is performed by an external diagnostic laboratory with results staying within 2.5 x 10^2^–1.5 x 10^4^ cfu mL^-1^. Our routine monitoring showed the presence of the agents *Pseudoloma neurophilia*, *Mycobacterium spp*., *Mycobacterium abscessus*, *Mycobacterium chelonae*, *Mycobacterium peregrinum*, and *Myxidium streisingeri* during the last 12 months prior to the study. During the study, the additional examination of colony animals was largely dispensed with, due to the large number of fish tested during the experiment.

### Samples

Over a period of 6 months, the fish colonies from all systems were carefully screened to identify fish showing clinical signs of disease. The inspection was carried out at least once a month by competent veterinarians. The daily inspection was carried out by the animal keepers who immediately reported suspect fish to the responsible veterinarians. Diseased zebrafish were caught from a tank and euthanized individually using a freshly made solution of tricaine methane sulfonate (MS-222; overdose 400 mg L^-1^) buffered with sodium bicarbonate. Fish were left in the solution for at least 10 minutes following cessation of opercular movement and absence of reaction to tactile stimuli, before being transferred to 50 mL centrifuge tubes using sterile, individually-wrapped forceps and stored at -20°C until shipment. In conjunction, 1 L of tank water (from the same tank as above) from the middle of the free water column was collected using sterile 1 L plastic buckets. 25 mL of detritus collected from the bottom of the tank with a sterile pipette were added to the water sample. This environmental sample was then passed through a sterile 0.2 μm filter (Nalgene Sterile Analytical Filter Unit, Thermo Fisher Scientific) under vacuum conditions using a water jet pump. The filter membrane containing all non-filtered contents of the sample was transferred to a 50 mL centrifuge tube and stored at -20°C until shipment.

### Analysis

Frozen fish and environmental samples were stored in 50mL centrifuge tubes and sent to IDEXX Laboratories, Inc. (Westbrook, ME, USA). Samples were tested for *Edwardsiella ictaluri*, *Flavobacterium columnare*, *Ichthyophthirius multifiliis*, Infectious spleen & kidney necrosis virus (ISKNV), *Mycobacterium spp*., *Mycobacterium abscessus*, *Mycobacterium chelonae*, *Mycobacterium fortuitum*, *Mycobacterium haemophilum*, *Mycobacterium marinum*, *Mycobacterium peregrinum*, *Myxidium streisingeri*, *Piscinoodinium pillulare*, *Pleistophora hyphessobryconis*, *Pseudocapillaria tomentosa*, and *Pseudoloma neurophilia* using real-time PCR. From 2019 on, the agents *Mycobacterium gordonae*, *Mycobacterium saopaulense* and Zebrafish picornavirus (ZfPV-1) were added to this panel and the genus-specific PCR for *Mycobacterium spp*. was no longer reported. Total nucleic acids were extracted with standard protocols using a commercially available platform (NucleoMag VET, Macherey-Nagel, Inc., Bethlehem, PA, USA). PCR testing for infectious agents were based on the IDEXX BioResearch proprietary service platform (IDEXX Laboratories, Inc., Westbrook, ME, USA) with all assays having an analytical sensitivity of 1–10 copies per PCR reaction. Briefly, all microbes were detected with real-time PCR assays using hydrolysis probes. A hydrolysis probe-based real-time PCR targeting prokaryotic and eukaryotic housekeeping genes (16S rRNA and 18S rRNA) was used to confirm nucleic acid integrity and ensure the absence of PCR inhibitors in the test sample. Real-time PCR was performed with standard primer and probe concentrations using a commercially available mastermix (LC480 ProbesMaster, Roche Applied Science, Indianapolis, IN, USA) on a commercially available PCR platform (Roche LightCycler 480). The data were analyzed with McNemar's test, a statistical test used on paired nominal data, using R 3.5.1 (R Foundation for Statistical Computing, Vienna, Austria). A minimum number of six positive PCR results are required when using this test at a significance level of 5% in order to obtain statistically significant results. Descriptive evaluation was performed if less than six positive PCR results were obtained for an individual pathogen.

## Results

### Signs of disease

A total of 49 fish from different genetic backgrounds showing clinical signs of severe illness were found in a period of six months and were euthanized for real-time PCR analysis. On two occasions two diseased fish were taken from the same tank simultaneously; whereas, in all other occasions, fish originated from separate tanks. 48 diseased fish were already older than two years. As a general rule, fish over the age of 18 months are euthanized as they are a reservoir for pathogens, but several strains of fish need to be aged for aging studies at the center. In summary, the following abnormalities were found in these animals: uncoordinated swimming behavior, emaciation, dropsy, deformed spine, exophthalmos, wounds, bleeding on the body surface, tumors, bleeding in the intestines, raised scales, coverings on the fins as well as color changes such as reddish or dark spots on the scales.

### Real-time PCR results

**Fig 1:**
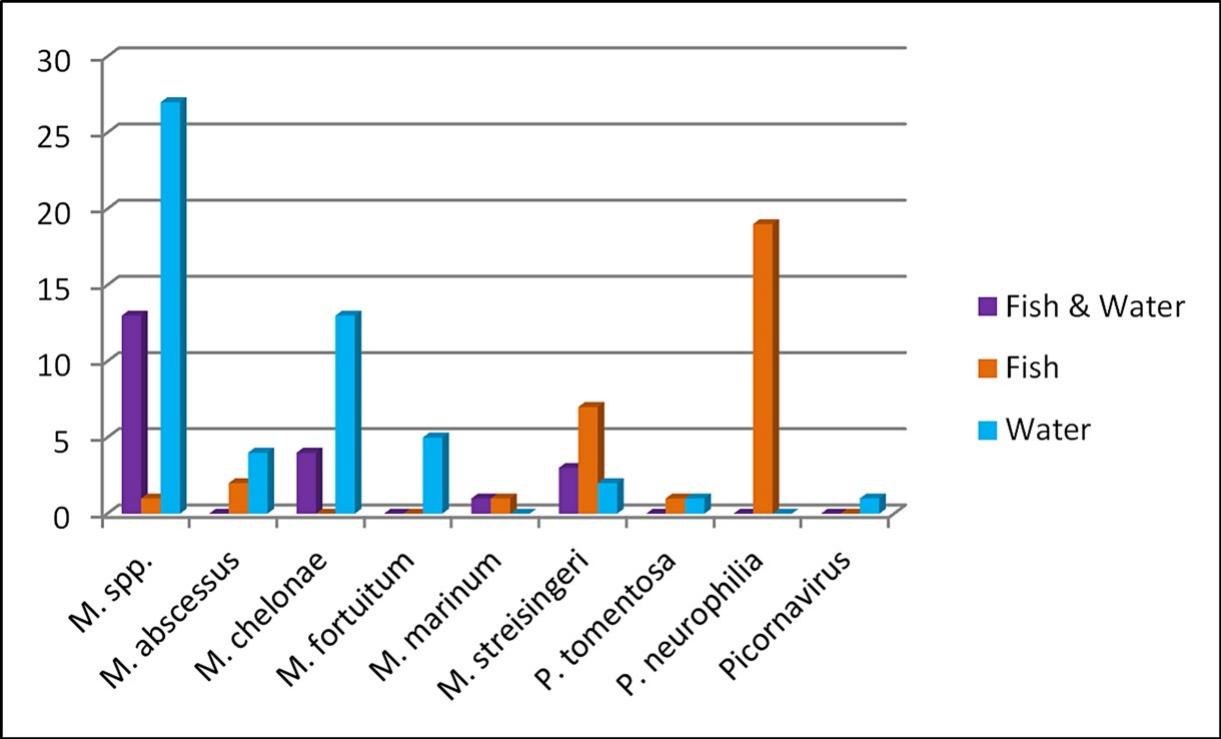
Number of fish and environmental samples (1L water and 25 mL detritus sterile filtered) that tested positive for various zebrafish pathogens using real-time PCR assays.

In 33% (16/49) of the examined frozen fish, none of the tested pathogens were detected. In contrast, only 8% (4/49) of the environmental samples consisting of the dry sludge generated by filtration of water and detritus showed a negative result. Only one pathogen was detected in 37% (18/49) of the cases, and in 31% (15/49) of the cases two or more pathogens were detected analyzing the frozen fish. Considering the water samples, 47% (23/49) tested positively for one pathogen, and 45% (22/49) tested positively for at least two pathogens. In summary, different pathogens were isolated by the two methods used (Fig 1).

Mycobacteria were partially present in both sample types. However, they were more frequently detected in the environmental samples. Genus-specific *Mycobacterium spp*. PCR revealed positive results in 13 cases using both sample types, in one case in the fish and 27 times in dry sludge samples only, indicating that detection of this pathogen in the water is significantly (p < 0.0001) more likely than in the fish. Similar results were obtained in the species-specific *M*. *chelonae* assay (p = 0.0009) where 4 out of 17 positively tested environmental samples matched with a positive fish PCR. Solely in two cases, fish samples tested positive for *M abscessus*, with the pathogen not being confirmed in the water samples. On the other hand, four water samples tested positive with negative fish PCR results (p = 0,68). In contrast to the previous agents, other pathogens were detected predominantly or exclusively in the frozen fish. An infection with the myxozoan parasite *M*. *streisingeri* was determined in 12 different tanks. Of these, three cases tested positive in both sample types, and two and seven times only tested positive in the water or fish samples, respectively (p = 0.18). The difference according to the sample type was most obvious in *Pseudoloma neurophilia*, which was found exclusively in fish (19/49) but never in environmental samples (p < 0.0001). Since less than six positive PCR results were obtained per pathogen, a descriptive evaluation could only be performed for *M*. *fortuitum*, *M*. *marinum*, *P*. *tomentosa*, and Picornavirus. *M*. *fortuitum* was detected in environmental samples only (5/49). *M*. *marinum* was found once in both sample types and once in the fish sample only. *P*. *tomentosa* was detected twice, once in a frozen fish and once in an environmental sample. Zebrafish picornavirus was detected only once in an environmental sample and never in a fish sample.

## Discussion

Accurate knowledge of the health status of laboratory fish is an essential part of good study design. Like for rodents, the examination of diseased aquatic animals should be an important part of any hygiene concept [24], as the probability of detecting infectious agents is highest here. The analysis of environmental samples, instead of the animal itself, has previously been described in various publications for rodents as well as for fish [11, 15, 17–22]. In this study, we examined whether individual real-time PCR analysis of diseased fish, PCR analysis of water samples including detritus from affected tanks, or a combination of both sample types was appropriate to reliably detect infectious agents. Equivalent to recent results obtained in rodent facilities [17], health monitoring programs of fish facilities performed solely by water analysis would satisfy the 3Rs. We showed that no single method is sufficient to cover all pathogens present and that a combination is essential. Our results are similar to those obtained by Crim et al., who compared detection of pathogens in frozen zebrafish, detritus, filtered water, and feces [21]. Our study differs, however, in the type of aquatic system used. Our samples were collected in a recirculating system routinely used in experimental fish facilities. We assume that pathogen concentrations in recirculating systems are lower because pathogens are rinsed out when tanks are flushed permanently. This is probably why microsporidium *Pseudoloma neurophilia* was never detected in environmental samples, but was frequently detected in fish. However, Crim et al. described inconsistent detection in a 1 L tank water sample. Another difference consists in the fact that water and detritus were not analyzed individually, but pooled and that additional real-time PCR assays for new pathogens such as *M*. *streisingeri* and ZfPV-1 were included in the test panel. No clear conclusion can be drawn for *Pseudocapillaria tomentosa*, as this pathogen was only detected once in a single fish and once in a water sample. DNA of this nematode has only been detected in our quarantine barrier. Lines are transferred from there into the main aquatic systems via embryo bleaching. Therefore it might be possible that *P*. *tomentosa* is either not present in our research barriers or that the prevalence is very low. Nevertheless, nematode eggs can potentially be shed together with fish eggs, and the effect of bleach solutions (25 to 50 ppm) typically used to surface-sanitize zebrafish embryos on *P*. *tomentosa* eggs is not known [25]. *P*. *tomentosa* eggs measure about 30 to 50 μm [6] and infected fish pass them with their feces. Eggs remain at the bottom of the tank due to their high density which is probably the reason why they can be better detected in detritus than in water samples [21, 22].

The evaluation of genus- and species-specific mycobacterial PCR assays provided a much clearer result, showing that detection of these bacteria in the environmental samples is more efficient than testing zebrafish directly. Mocho et al. demonstrated that detection via sump swabs was superior to analysis of numerous fish using PCR technology despite a smaller number of samples [11]. Due to the long incubation period of mycobacteria, their detection in fish might be considerably delayed [26]. The presence of identical *Mycobacterium* species both in fish and biofilms suggests that transfer from the environment to fish may occur, for example when fish feed on tank detritus [4, 26]. Whether mycobacterial infection is present and may influence fish health cannot be confirmed by PCR alone. Instead, histopathological analysis is necessary, which was not part of this study [11].

An alarming finding was the detection of *Mycobacterium marinum*, which has never been detected in our facility in recent years either during routine monitoring or during examination of diseased fish. The first case (a positive result in both the fish and environmental sample) was from a quarantine room, and the second case (only the fish tested positive) was from one of the experimental aqua systems a short while after. *Mycobacterium marinum* is a zoonotic pathogen that can infect the skin and lead to granulomatous lesions that can progress to tenosynovitis, arthritis, and osteomyelitis, even in immunocompetent humans [8]. Therefore, intensive retesting has been carried out to estimate its spread within the aqua systems. Although the presence of *Mycobacterium marinum* was confirmed a second time in water from the affected tank two weeks after the first result, no spread to other tanks was indicated up to the publication of this article. The findings induced the provision of additional personal protective equipment, such as gloves covering the entire arm and face shields. Mycobacteria are able to form resistant biofilms, making disinfection a challenge [5]. After all strains were transferred to a new aqua system in another building using an adequate egg bleaching protocol [5] and additional testing of larvae at day 6, the quarantine unit was finally shut down for restoration.

In addition to the pathogens mentioned above, two infective agents recently described for zebrafish, *Myxidium streisingeri* and Zebrafish Picornavirus 1 were tested and detected in environmental samples for the first time in our facility. *Myxidium streisingeri* is a myxozoan parasite of the ducts associated with the kidney [27], and the Zebrafish Picornavirus 1 (ZfPV-1) is associated with gut epithelia infections [28]. Whether these agents affect research outcomes in wildtype or mutant zebrafish lines remain to be determined. As with other unwanted agents, these infections should be considered as possible underlying causes of unexpected variations in research [2]. Based on the preliminary results, detection of *M*. *streisingeri* in fish seems to be more reliable than in the water, although the number of observations still is too low to form a concrete conclusion. As PCR assay for ZfPV-1 was applied only later in the study, the single result for this pathogen is not yet sufficient to provide a recommendation.

In conclusion, our experiments underline the need to use multiple sample types to detect all pathogens in fish populations, which is in close consensus with other publications. In addition to sentinel fish used for routine monitoring, swab samples, total bacterial counts of system water, and the analysis of diseased fish are important parts of a reliable hygienic monitoring concept. We suggest that facilities monitor not only diseased fish but also healthy colony animals and environmental samples like water or detritus by PCR. Otherwise, pathogens might be systematically overlooked such as *Pseudoloma neurophilia*, found exclusively in fish samples, or various mycobacteria, predominantly present in the environment. In our study, at least one pathogen could be detected in 47 out of 49 occasions by combining both sample types. However, since the prevalence of agents was unknown, no information can be provided about how often pathogens have been overlooked by both sample types. Further studies are necessary for those pathogens that were not detected in sufficient numbers in this study in order to be able to perform a statistical evaluation and to recommend a reliable monitoring strategy. If pathogens such as mycobacteria are found in the environment only, further tests, such as a histopathological analysis, are advisable to ensure that the fish are infected. However, information about agents present in the environment is very important in order to be able to take appropriate action at an early stage. Detection of an agent does not necessarily mean exclusion from experiments. Depending on agent and research topic, possible influence on results must be assessed case-by-case on the basis of available literature.
